# Extraction of polysaccharides from *Codonopsis pilosula* by fermentation with response surface methodology

**DOI:** 10.1002/fsn3.1958

**Published:** 2020-10-30

**Authors:** Shuai Yuan, Chang‐Yuan Xu, Jie Xia, Yan‐Ni Feng, Xi‐Feng Zhang, You‐Yu Yan

**Affiliations:** ^1^ College of Biological and Pharmaceutical Engineering Wuhan Polytechnic University Wuhan China; ^2^ College of Veterinary medicine Qingdao Agricultural University Qingdao China

**Keywords:** antioxidation, codonopsis, polysaccharide, response surface method

## Abstract

*Codonopsis pilosula* is a kind of traditional Chinese medicine used to treat weak spleens, stomach problems, anemia, and fatigue. Polysaccharide is one of main components of *Codonopsis pilosula*. In this study, response surface methodology (RSM) was used to optimize the extraction parameters of *Codonopsis pilosula* polysaccharides (CPP) by fermentation. The exaction temperature (°C), yeast liquid volume (2 mg/ml, ml), and time (h) were employed effects. Results indicated that the best extraction conditions were the following: extraction temperature 24.75°C, yeast liquid volume 2.96 ml (5.92 mg), and a fermentation time of 21.03 hr. After purification with DE52 and Sephadex G‐100, the molecular structure was determined by ultraviolet–visible (UV) spectroscopy, Fourier transform infrared (FTIR) spectroscopy, and nuclear magnetic resonance (NMR) (^1^H and ^13^C). The monosaccharide composition of CPP1 was determined to be mannose (1.76%), glucose (97.38%), and arabinose (0.76%). CPP1 exhibited high antioxidant activities in scavenging ABTS radicals, ferreous ions, and superoxide ion radicals. Thus, CPP1 could be used as an antioxidant or functional food.

## Graphical abstract

Response surface methodology (RSM) was used to optimize the extraction parameters of *Codonopsis pilosula* polysaccharides (CPPs) by fermentation.

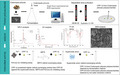

## 1 INTRODUCTION

*Codonopsis pilosula* (*C. pilosula*) is in the family Campanulaceae, its root is a commonly used Chinese medicine, and it is mainly planted in China, Japan, and Korea (Chen & Huang, 2018; Liu et al., 2018). As a traditional Chinese medicine, *C. pilosula* has been widely recorded in many ancient books. It has been mainly used for strengthening the spleen, moistening the lung, nourishing blood, engendering fluid, enhancing immune function, and modulating antitumor effect (Bai et al., 2018; Zou et al., 2014).

At present, the commonly used polysaccharide extraction methods include hot water extraction, acid–base extraction, and ultrasonic‐assisted extraction (Dou et al., 2019; Yilmaz & Sebnem, 2017). These methods have their advantages and disadvantages. We use yeast to grow and use the characteristics of oligosaccharides such as glucose and fructose. After fermenting *C. pilosula*, the yeast consumes monosaccharides and disaccharides in *C.pilosula* to improve the extraction efficiency and purity of polysaccharides and explore a new type of polysaccharide extraction method.

In this study, RSM was employed to estimate the influence of different extraction parameters (fermentation temperature, time, and yeast addition) on the yields of polysaccharides from *C. pilosula*. Column chromatography was used to purify water‐extracted polysaccharides and fermentation polysaccharides. Then, NMR, FTIR, high‐performance liquid chromatography (HPLC), and scanning electron microscopy (*SEM*) were employed to characterize the polysaccharide. *SEM* is an electron microscope developed after TEM. *SEM* is also one of the main instruments of microstructure analysis, which has been widely used in materials, metallurgy, minerals, biology, and other fields. However, *SEM* can only observe the micromorphology of the material surface and cannot obtain the information of the material interior. Furthermore, the antioxidant activities of polysaccharides were evaluated, including the metal iron ion scavenging ability, ABTS radical scavenging ability, and superoxide anion radical scavenging activity.

## 2 MATERIALS AND METHODS

### 2.1 Materials

*C. pilosula* was purchased from the local market in Hubei, China. The DEAE‐52 cellulose and Sephadex G‐100 were obtained from Solarbio (Beijing China). All chemicals were analytically pure grade.

### 2.2 Extraction of polysaccharide

*C. pilosula* was washed with distilled water and dried in the sun. Then, the dry *C. pilosula* was ground into powder. Then, to a 250‐ml shake flask, 1 g *C. pilosula* powder, 50 ml LB medium(peptone 10 g/L, yeast extract 5 g/L, NaCl 5 g/L, pH = 7), and 3 ml yeast sample solution (the beer active dry yeast from Angel Yeast Company and distilled water were mixed at the concentration of 2 mg/ml) were added. The sample was placed in the shaker （24.5°C, 200 rpm） for 21 hr. The fermentation broth was suction filtered to obtain an extract. The obtained extract was poured into a macroporous resin elution column for decolorization treatment, and using sevage solution (chloroform:n‐butanol, 4:1) protein was removed by separating with a separatory funnel. The supernatant was precipitated with fourfold volumes of 95% ethanol at 4°C for 16 hr. Precipitate was collected by centrifugation (2397 *g*, 10 min) and lyophilized to obtain crude polysaccharides, that is, CPP.

Measure 0, 0.2, 0.4, 0.6, 0.8, 1.0 ml of 0.1 g/L glucose standard solution (Accurately weigh 0.1 g glucose, add 100 ml distilled water to dissolve, and dilute to volume in a volumetric flask 100 ml) into the test tube, add distilled water to make the volume up to 1.0 ml in the volumetric flask, and then add 0.5 ml of 6% phenol (Accurately weigh 3 g of phenol, add 50 ml of distilled water to dissolve, and dilute to 10 ml in a 10 ml volumetric flask) solution And 2.5 ml concentrated sulfuric acid, heated in boiling water bath for 30 min, and finally measured the absorbance of the sample at 490 nm (Masuko et al., 2005）. A glucose standard curve obtained with regression equation: *y* = 14.25*x* − 0.0599 (*R* ^2^ = .998), where y is absorbance at 490 nm and *x* is glucose concentration (mg/ml). The standard curve had a good linear relationship between 0.01–0.1 mg/ml.

Three single factors (fermentation temperature (°C), time (h), and yeast addition (ml)) were used for further extraction of CPP（Cai,et al., 2019）. As shown in Table 1, on the basis results of single factor experiment, Box–Behnken design (BBD)‐RSM were used to investigate the yield of CPP (Table 1). An the polysaccharide yield of the experimental group based on the curve of glucose standard solution. The parameters of the model were estimated by the least square method through 17 measurement experiments, and then the model is established (Table 2). The absorbance at 490 nm of sugar by the phenol sulfuric acid method was used as the detection index. By applying multiple regression analysis to the experimental data, the response variable and the test variables were related by the second‐order polynomial equation. The obtained test data were used for multiple regression fitting and optimization of process parameters by using Design‐Expert 8.0 software. The best fermentation conditions were used for verification and detection, and compared with the ideal value of the software.

**TABLE 1:** Levels and code of extraction variables used in Box–Behnken design

Variable	Coded levels		
	−1	0	1
Exaction temperature (X_1_,°C)	20	25	30
Yeast liquid volume (X_2_, ml)	2	3	4
Exaction time (X_3_, h)	16	20	24

**TABLE 2:** Box–Behnken experimental design and the results for extraction yield of CPP

Run	X_1_	X_2_	X_3_	Absorbance	Yield (mg)
1	30	3	24	0.541	16.561
2	25	4	24	0.533	16.286
3	20	2	20	0.534	16.320
4	25	3	20	0.565	17.387
5	20	3	24	0.551	16.905
6	25	2	24	0.535	16.355
7	20	4	20	0.520	15.838
8	25	2	16	0.527	16.079
9	25	3	20	0.569	17.525
10	25	3	20	0.560	17.215
11	25	3	20	0.563	17.319
12	30	2	20	0.522	15.907
13	30	4	20	0.527	16.079
14	25	4	16	0.522	15.907
15	20	3	16	0.534	16.320
16	30	3	16	0.544	16.664
17	25	3	20	0.561	17.250

### 2.3 Preparation of polysaccharides

CPPs (80 mg) were redissolved in ultrapure water (4 ml), and DEAE column (I.0 × 40 cm) was used for further classification. Gradient elution was carried out with 0, 0.05, 0.1, 0.2, 0.3, and 0.5 mol/L NaC1 solution at a flow rate of 1 ml/min, with 5 ml in each tube (Jin et al., 2016). The sugar of each tube was determined at 490 nm by the phenol sulfuric acid method. The major peak polysaccharide (CPP1) was concentrated and further purification through a column (1.0 × 40 cm) of Sephadex G‐100 at a flow rate of 0.5 ml/min. And then the purified polysaccharide fractions (CPP1) were analyzed, concentrated, and freeze‐dried.

### 2.4 UV spectroscopy and FTIR spectroscopy

The CPP1 sample solution was set to 0.1 g/ml, and full wavelength scanning was performed at 190–800 nm. Based on the spectrum, it was inferred whether the obtained polysaccharide component had free nucleic acids and proteins. CPP1 was mixed with spectrum grade potassium bromide powder and then milled into pellets for infrared spectrum determination. According to the previously reported method, FTIR spectra were recorded from 4,000 to 400 cm^−1^ by FTIR spectrometer (tensor 27, Bruker, Karlsruhe, Germany) (Jouraiphy et al., 2008).

### 2.5 Monosaccharide composition analysis

The monosaccharide composition of CPP1 was determined by PMP precolumn derivatization HPLC (Cai et al., 2018). The hydrolyzed CPP1(10 mg) or monosaccharide standard solution (50 μl) was mixed with 0.6 mol/L sodium hydroxide (50 μl), and then treated with a 0.5 mol/L methanolic solution of PMP (100 μl) at 70°C for 100 min. The product was neutralized with 100 μl hydrochloric acid (0.3 M), and distilled water was added to 1 ml. And then the reaction product was made up to extracted with 2 ml chloroform three times. Filtered the solution through 0.45 μM filter membrane and carried out monosaccharide composition analysis by HPLC.

### 2.6 *SEM* analysis

*SEM* (SU8010, Hitachi High‐Technologies Co., Tokyo, Japan) was employed to observe the molecular morphologies of CPP1. In brief, the CPP1 powder was placed on the sample stage and coated with gold powder, and the *SEM* images were observed under the high vacuum condition.

### 2.7 NMR analysis

The ^1^H and ^13^C NMR spectra of CPP1 were recorded using a Bruker AV‐400 NMR spectrometer (Bruker Instrumental Inc., Billerica, Massachusetts, USA) at 25°C, and the CPP1 was dissolved in dimethyl sulfoxide (DMSO).

### 2.8 Antioxidant activity analysis

The superoxide anion radical scavenging activity, ABTS radical scavenging activities, and ferrous iron ion scavenging ability of CPP1 were determined based on previously reported methods (Carter, 1971; Finkel et al., 2000; Peng et al., 2017; Re et al., 1999; Wang, Wang, et al., 2016; Wang, Ding, et al., 2016; Zhang et al., 2018; Zhu et al., 2018). 1, 2, 4, 8, and 10 mg/ml of CPP1 were dissolved in water.

#### 2.8.1 Superoxide anion radical scavenging activity

7 mmol/L pyrogallol aqueous solution was prepared in advance. Take 0.3 ml of the prepared polysaccharide solution, add 0.9 ml of Tris‐HCl, 0.09 ml of pyrogallol, and 0.3 ml of 10 mol/L concentrated hydrochloric acid, respectively, and then react at room temperature for 20 min. Then, the absorbance A_1_ was determined at 420 nm. The absorbance of ultrapure water replacing polysaccharide solution is A_0_ and that of ultrapure water replacing chromogenic solution is A_2_.

#### 2.8.2 ABTS radical scavenging activities

Accurately transfer 400 μl of the prepared polysaccharide solution into a 2 ml centrifuge tube, add 1,600 μl of diluted ABTS stock solution, react at room temperature for 10 min, detect its absorbance at 734 nm, and record it as A_1_. Accurately transfer 400 μl of each prepared polysaccharide solution, place 2 ml centrifuge tube, add 1,600 μl distilled water into each tube, react at room temperature for 10 min, and record the absorbance value as A_2_. Absorb 400 μl distilled water, add 1,600 μl ABTS stock solution, react at room temperature for 10 min, and record the absorbance value as A_0_.

#### 2.8.3 Ferrous iron ion scavenging ability

Transfer 400 μl of the prepared polysaccharide solution, 80 μl phenanthroline test solution, 40 μl FeCl_2_ solution and 1.08 ml distilled water were added in order to react at a constant temperature for 2 hr. The absorbance was determined at 562 nm and recorded as A_1_. 400 μl of distilled water instead of the sample, the absorbance was determined at 562 nm and recorded as A0. Measure 400 μl polysaccharide solution, add 80 μl phenanthrazine test solution and 1.12 ml distilled water in order to react at constant temperature for 2 hr, and then determine the absorbance as A_2_.

The superoxide radical scavenging activity was calculated according to the following equation: Scavenging activity (%) = [1 − (A_1_ − A_2_)/A_0_] × 100%. The experiment was repeated three times with duplicate samples.

## 3 RESULTS AND DISCUSSION

### 3.1 Response surface analysis

Based on single factor investigation results, 17 runs of Box–Behnken test factors design and results of test factors are shown in Table 2. The results showed that the absorbance of CPP varied from 0.520 to 0.569, and the optimization conditions for extraction of CPP were as follows: extraction temperature of 24.75°C, yeast liquid volume of 2.96 ml (5.92 mg beer active dry yeast), and a fermentation time of 21.03 hr. The quadratic regression equation with the absorbance of CPP as the objective function was obtained:

The *F*‐test and P‐values were used to measure the significance of the coefficients of the model. As shown in Table 3, X_1_X_2_ and X_1_X_3_ were significant; X_3_ was highly significant. These data indicate that the model established by the experiment was feasible. The precision value of 19.927 indicates that the model can predict experimental results. An *R* ^2^_Adj_ value of .9703 indicates that the model can prove the prediction of 97.03% of the response value, and the determination coefficient *R* ^2^ of .9870 indicates that the model has a good degree of fit, and the absorbance value of the polysaccharide of *C. pilosula* can be analyzed and predicted. The *R* ^2^_Pred_ being equal to .9578 is not significantly different from the *R* ^2^ of .9870, indicating that there was no need to further optimize the response surface equation.

**TABLE 3:** Analysis of variance of the experimental results of the BBD in extraction of CPP

Variables	Sum of squares	*df*	Mean square	*F*‐value	*p*‐Value
Model	0.004401932	9	0.000489104	59.08067581	<.0001
X_1_	3.125E‐06	1	3.125E‐06	0.377480587	.5584
X_2_	3.2E‐05	1	3.2E‐05	3.865401208	.0900
X_3_	0.000136125	1	0.000136125	16.44305436	.0048
X_1_X_2_	9.025E‐05	1	9.025E‐05	10.90163934	.0131
X_1_X_3_	0.0001	1	0.0001	12.07937877	.0103
X_2_X_3_	2.25E‐06	1	2.25E‐06	0.271786022	.6182
X_1_^2^	0.000637011	1	0.000637011	76.94691431	<.0001
X_2_^2^	0.002748642	1	0.002748642	332.0188911	<.0001
X_3_^2^	0.000326063	1	0.000326063	39.38640389	.0004
Residual	5.795E‐05	7	8.27857E‐06		
Lack of fit	6.75E‐06	3	2.25E‐06	0.17578125	.9076
Pure error	5.12E‐05	4	0.0000128		
*R* ^2^ = .9870	*R* ^2^_Adj_ = .9703	*R* ^2^_Pred_ = .9578	CV = 0.53%	Adeq Precision = 19.927	

**FIGURE 1:**
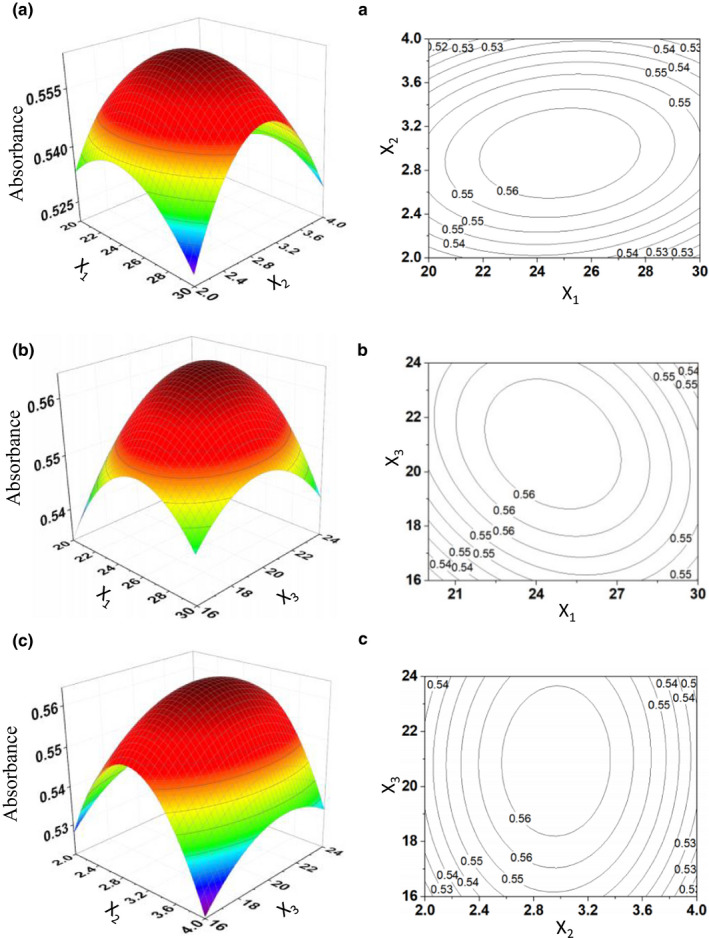
Response surface plots showing effects of variables on the extraction yield of CPP. A(a). The response surface of the effect of exaction temperature (X_1_, °C) and yeast liquid volume (X_2_, ml). B(b). The response surface of the effect of extraction temperature (X_1_, °C) and exaction time (X_3_, h). C(c). The response surface of the effect of the yeast liquid volume (X_2_, ml) and exaction time (X_3_, h)

Design‐Expert (version 8.0) was used to create the relationship between the independent and dependent variables, and the 3D response surface and contour plots are shown in Figure 1. Figure 1A and a shown the response surface and contour map of X_1_ and X_2_ to the yield of CPP. The influence of each factor in the response surface is staggered, and the degree of its influence can be clearly reflected by the intensity of the contours in the contour map and the steep angle in the response surface. The denser contours indicate a steeper response surface and higher impact. The larger spacing between contours indicates a smaller impact. It can be seen in Figure 1 that the above three factors have an important impact on the results.

In this experiment, the optimal extraction conditions of CPP were optimized by using RSM. At this time, the predicted absorbance value was 0.5642. The results of repeatability verification experiment were 0.552 (16.898 mg), 0.551(16.868 mg), 0.557(17.052), 0.550 (16.837), and 0.579(17.725). The average absorbance value of the experimental experiment was 0.5780, and the relative error was only 2.45%. Therefore, this model is reliable and can be used to optimize the extraction process of *C. pilosula* polysaccharides.

### 3.2 Separation and purification of polysaccharides

**FIGURE 2:**
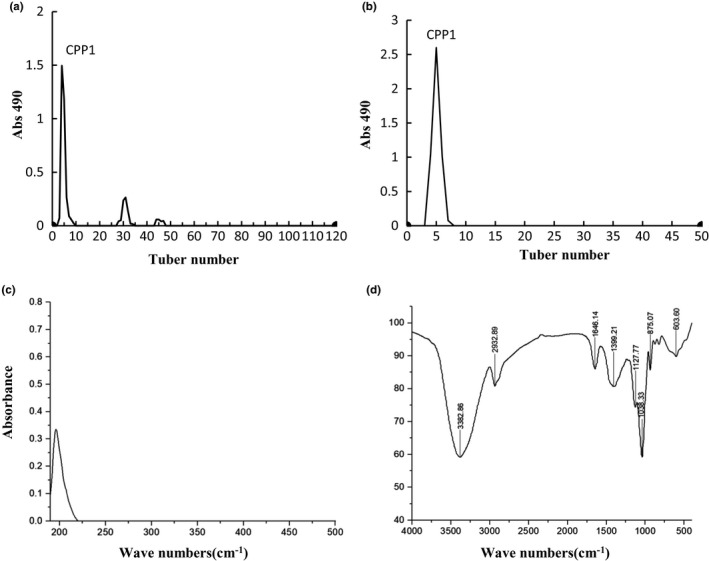
DEAE‐52 anion exchange chromatography (a) and Sephadex G‐100 size‐exclusion chromatography (b) of CPP; UV spectrum of CPP1 (c) and FTIR spectrum of CPP1 (d)

As shown in Figure 2a and b, crude CPPs showed three peaks after the purification of DE‐52, and the major peak was CPP1 (Figure 2a). Then, CPP1 was unimodal, symmetrical, and peaked in Sephadex G‐100 column chromatography (Figure 2b). Therefore, we obtain uniform and stable sample of CPP1.

### 3.3 UV analysis and FTIR spectroscopy

As shown in Figure 2c, no absorption peak of CPP1 was detected at about 260 and 280 nm in the UV analysis in indicating that CPP1 was free of nucleic acids and protein. The FR‐IR spectrum of CPP1 is shown in Figure 2d (Jin et al., 2018). The absorption bands within the ranges 3,600–3,000, 3,000–2,800, 1,400–1,200, and 1,200–700 cm^−1^ were the characteristic absorption peaks of polysaccharides. The broad band at 3,382.86 cm^−1^ was the characteristic band of O–H tensile vibration, and the signal at 2,932.89 cm^−1^ was C–H tensile vibrations. The relatively strong absorption peak at 1,646.14 cm^−1^ indicated the existence of C–O bond, and the absorption peaks at 1,399.21 cm^−1^ was attributed to –CH_2_– shear vibration. The peak value in the range of 1,300–1,000 cm^−1^ was the characteristic of carbohydrates. In addition, the peak value of CPP1 at 875.07 cm^−1^ indicated that the type of sugar bond was β‐type glycosidic bonds. The results indicated that CPP‐1 possessed typical peaks of polysaccharide absorption.

### 3.4 NMR analysis

**FIGURE 3:**
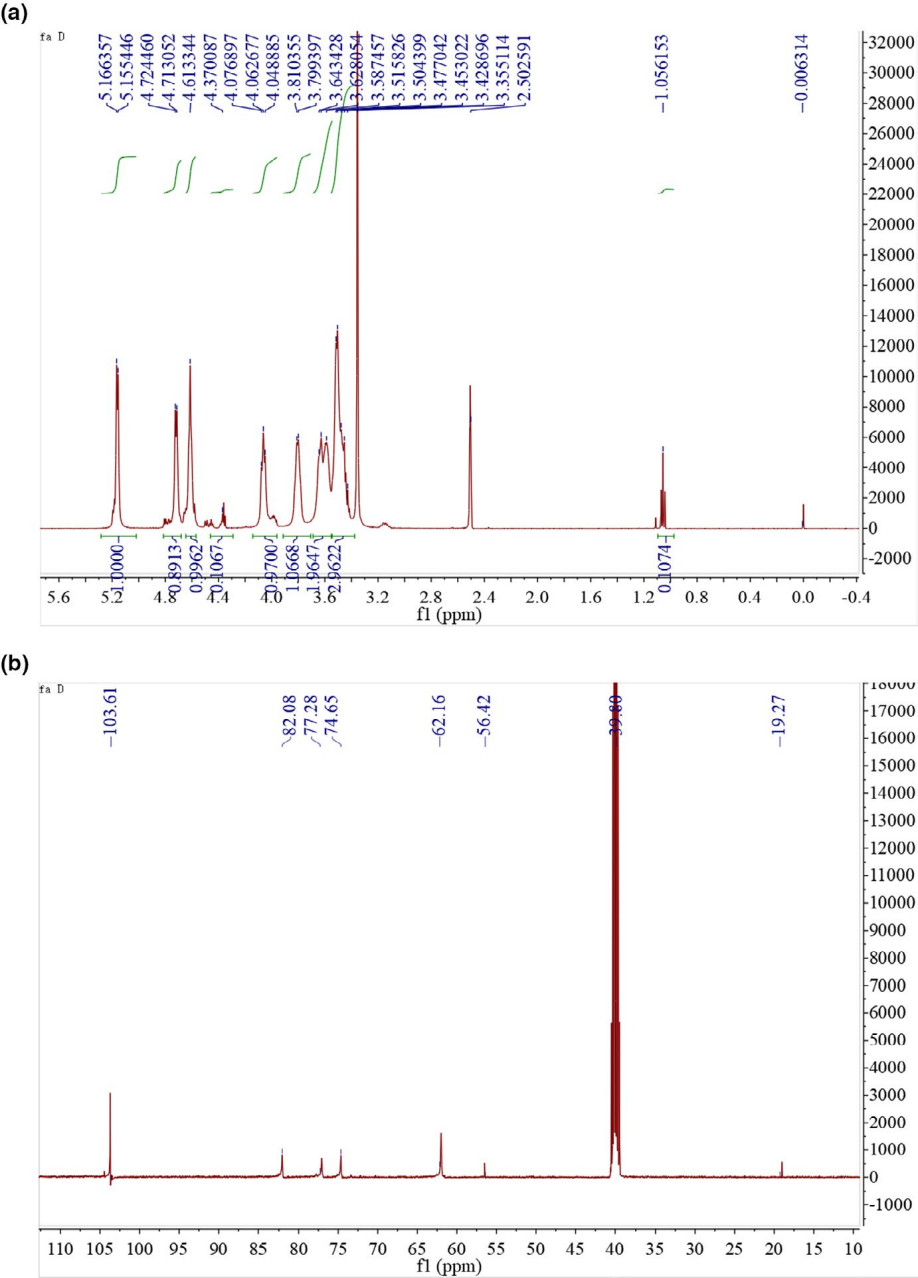
The ^1^H (A) and ^13^C spectra of CPP1 in DMSO at 25°C

The structures of CPP1 was further analyzed by NMR. The ^1^H NMR spectra showed that the chemical shifts of CPP1 were mainly between 3.5 and 5.3 ppm (Zhu et al., 2011). As shown in Figure 3a, the signals in the range of 4.3–4.7 ppm in the ^1^H NMR spectrum indicated that CPP1 contained a β‐glycoside structure (Li et al., 2018). These results were consistent with the glycoside bond types of FTIR analysis. The weak peaks at 5.00–5.29 ppm were attributed to α‐Ara residues, and the peak at 4.90 ppm was attributed to α‐Glc residue (Li et al., 2017; Xu et al., 2016). In addition, signals between 3.62 and 3.35 ppm indicate the presence of methoxyl. As shown in the ^13^C NMR spectrum (Figure 3b), there were several isopropanol carbon signals in the range of 103.1 ppm, the signal near 19 ppm was expressed as methyl in acetyl, and the other non isopropanol carbons were appeared between 60.7 ppm to 80.9 ppm.

### 3.5 Morphological properties

**FIGURE 4:**
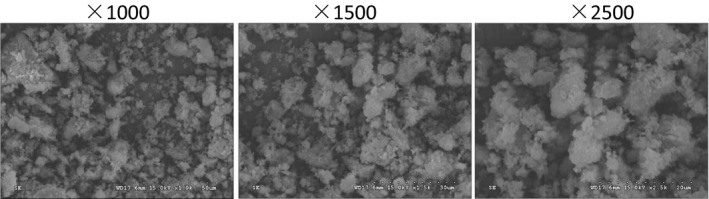
Scanning election microscope (*SEM*) photographs of CPP1

The *SEM* of CPP1 is shown in Figure 4. The polysaccharide has particle size and aggregates of irregular geometric shapes. The shapes and structures of polysaccharides may be influenced by product preparation or extraction and purification methods (Nep & Conway, 2010).

### 3.6 Monosaccharide composition analysis

**FIGURE 5:**
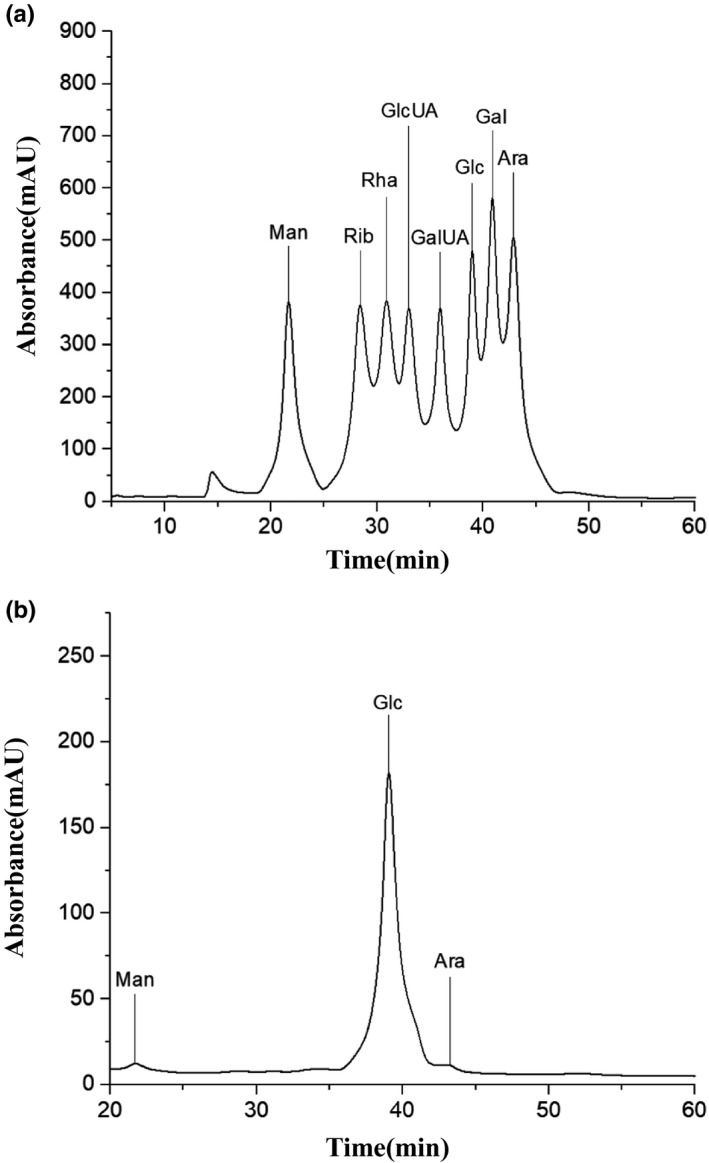
HPLC chromatograms of standard monosaccharides (a) and CPP1 hydrolysate (b)

Figure 5 shows the HPLC chromatograms of the release of PMP‐derived monosaccharides from CPP1 as well as those of eight standard monosaccharides. Based on the retention time of the derivatized monosaccharide standards, the monosaccharide composition of CPP1 was determined according to the retention time of the derived monosaccharide. CPP1 was mainly composed of mannose (Man), glucose (Glc), and arabinose (Ara), in the proportions of 1.76%, 97.38%, and 0.86%, respectively.

### 3.7 In vitro antioxidant activities of CPP1

Polysaccharides are important natural product resources. Studies have shown that active polysaccharides have a variety of biological activities, including antioxidant, anti‐inflammatory, hypoglycemic, and hypolipidemic. Researchers are very concerned about the antioxidant capacity of active polysaccharides, especially in vitro antioxidant activity (Ahmad et al., 2020; Munir et al., 2020). The antioxidant mechanism can be summarized as follows: elimination of active free radicals, elimination of inactive oxidation factors, binding of metal ions, and so on. There are many factors affecting the antioxidant activity of polysaccharides, which may include the source, chemical structure, molecular weight, purity, and spatial conformation. The antioxidant test methods of polysaccharides in vitro can be summarized as DPPH radical scavenging ability, ABTS activity free radical scavenging ability, oxygen free radical scavenging ability, superoxide anion radical scavenging ability, and metal ion chelating ability (Yao et al., 2020).

**FIGURE 6:**
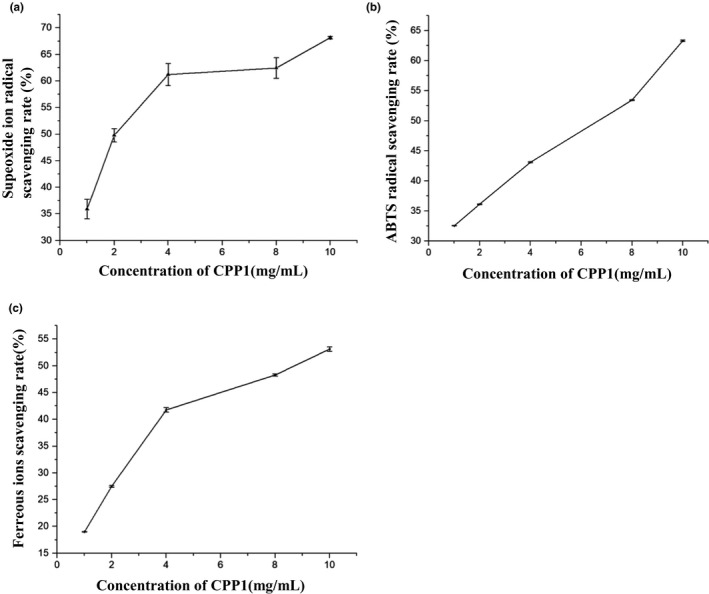
Antioxidant activity of CPP1. (a) Scavenging effects of CPP1 on superoxide ion radicals. Values are means ± *SD* (*n* = 3). (b) Scavenging effects of CPP1 on ABTS radicals. Values are means ± *SD* (*n* = 3). (c) Scavenging effects of CPP1 on metal ion radical scavenging rate. Value are means ± *SD* (*n* = 3)

Superoxide anion radicals are weak oxidants and therefore harmless to the body. However, excess superoxide radicals play an important role in the formation of secondary radicals such as hydrogen peroxide, hydroxyl radicals and singlet oxygen, which may lead to tissue damage. Superoxide radicals are the initial free radicals produced by the mitochondrial electron transport system. In addition, these free radicals can produce other strong free radicals, which may cause a variety of diseases (Vaz et al., 2011). Polysaccharides are a kind of highly effective free radical scavenger (Xiong et al., 2020). As shown in Figure 6a, CPP1 exhibited stronger superoxide anion radical scavenging activity. The maximum scavenging effect of CPP1 was 68.16 ± 0.23% at 10 mg/ml, which suggested that CPP1 was a good superoxide anion radical scavenger and protected the body from oxidative damage.

ABTS assay is usually used to determine the total antioxidant capacity of individual compounds or complex mixtures of various plants (Katalinic et al., 2006). ABTS radical scavenging capability was one of the methods to investigate the antioxidant activity of polysaccharides (Patel et al., 2018). As shown in Figure 6b, the concentration‐dependent free radical scavenging activity of CPP1 was researched. At the concentration of 10 mg/ml, CPP1 had a scavenging effect of 63.32 ± 0.12% on ABTS.

Metal chelating activity is generally considered as an antioxidant mechanism because it reduces the concentration of catalytic transition metals in lipid peroxidation (Chen et al., 2012). Ferric zinc is a sensitive reagent that can be mixed with ferrous ions to form a colored species (iron (II)–ferric zinc complex). After antioxidants are introduced to the system, they compete with iron and zinc for ferrous ions, reducing the absorbance of the solution. The polysaccharide has some metal ion scavenging activity (Medlej et al., 2020). As shown in Figure 6c, the different concentrations of CPP1 (1, 2, 4, 6, 8, and 10 mg/ml) showed certain metal chelating ability (12.03%‐44%).

## 4 CONCLUSIONS

RSM method was used to optimize the process parameters of CPP extraction by fermentation. The optimum extraction conditions were obtained: fermentation temperature of 24.57°C, yeast addition of 5.92 mg, and fermentation time of 21.03 hr. RSM provided a valuable method for optimizing CPP extraction process. CPPs were purified by DE‐52, and CPP1 was characterized by UV, FTIR, HPLC, NMR, and *SEM*. The results showed that CPP1 was mainly composed of mannose, glucose, and arabinose, with the proportions of 1.76%, 97.38%, and 0.76%, respectively. The characteristic FTIR absorption peaks of polysaccharides were observed in the infrared spectra of CPP1. CPP1 showed higher free radical scavenging activity against superoxide anions, ABTS, and iron ions. The results showed that CPP1 could be used as a natural antioxidant.

## 5 ETHICAL GUIDELINES

Ethics approval was not required for this research.

## CONFLICTS OF INTEREST

The authors declare no conflict of interest.

## AUTHOR CONTRIBUTIONS

Zhang XF and Yan YY designed the study. Yuan S, Xu CY, Xia J, and Feng YN collected data. All authors agreed the final version.

## Data Availability

Research data are not shared.
